# Assessment of Longitudinal Left Ventricle Deformation by 2-Dimensional Speckle Tracking Echocardiography Obtained from Different Views in Cats

**DOI:** 10.3390/vetsci7030104

**Published:** 2020-08-06

**Authors:** Domenico Caivano, Mark Rishniw, Lucia Baiona, Francesco Birettoni, Noemi Nisini, Francesco Porciello

**Affiliations:** 1 Department of Veterinary Medicine, University of Perugia, Via San Costanzo 4, 06126 Perugia, Italy; lucia.baiona@studenti.unipg.it (L.B.); noemi.nisini@gmail.com (N.N.); francesco.porciello@unipg.it (F.P.); 2 Department of Clinical Sciences, College of Veterinary Medicine, Cornell University, Ithaca, NY 14853, USA; mr89@cornell.edu

**Keywords:** echocardiography, feline, left ventricular function, strain, strain rate

## Abstract

Two-dimensional speckle tracking echocardiography (STE) is a novel, angle-independent imaging technique useful to assess myocardial function by strain and strain rate analysis in human and veterinary medicine. Commonly, the left apical four-chamber (LAP4Ch) view is used to assess left ventricular (LV) longitudinal deformation in dogs and cats. However, the right parasternal four-chamber (RP4Ch) view is often more easily obtained than the LAP4Ch view in cats. No studies exist comparing longitudinal strain and strain rate values using STE from different echocardiographic views in cats. Therefore, we examined the agreement between RP4Ch and LAP4Ch for assessment of LV longitudinal strain and strain rate in cats. We acquired 2D echocardiographic cineloops from RP4Ch and LAP4Ch views and analyzed LV longitudinal strain and strain rate in 50 cats (31 healthy cats and 19 cats with different disease states) using Xstrain^TM^ software. Peak systolic strain and strain rate values of endocardial and epicardial border were used for the analysis. The two echocardiographic views were compared using limits-of-agreement analyses and intra-observer measurement variability was assessed. We could obtain longitudinal strain and strain rate from the RP4Ch view in all cats. Strain, but not strain rate, had good intra-observer measurement variability (<10% vs. <20%). However, only endocardial strain values obtained with the two views agreed sufficiently to be used interchangeably (95% limits of agreement: −3.28, 2.58). Epicardial strain/strain rate and endocardial strain rate values did not agree sufficiently to be used interchangeably (95% limits of agreement: −11.58, 9.19; −2.28, 1.74; −1.41, 1.36, respectively). Our study suggests that RP4Ch view was feasible for assessment of the LV longitudinal deformation analysis by STE in cats, but only endocardial longitudinal strain values obtained from the two different views were interchangeable.

## 1. Introduction

Two-dimensional speckle tracking echocardiography (STE) is an advanced imaging technique useful to assess myocardial function by strain and strain rate analysis in human and veterinary medicine [1,2,3,4,5,6,7,8,9,10,11]. This novel echocardiographic modality assesses myocardial deformation using the standard 2-D images to follow the speckles (natural acoustic tissue reflections) frame-by-frame during the cardiac cycle [12,13]. Using STE, clinicians can evaluate myocardial deformation independent from translational cardiac movements and beam angle [1,12,13]. Several studies have focused on assessing the components of myocardial deformation of the left ventricle (LV), using the left apical four-chamber (LAP4Ch) view for longitudinal strain and strain rate and right parasternal short-axis views for radial and circumferential deformation in dogs and cats [4,5,6,7,14,15,16,17].

Recently, investigators have described the feasibility of assessing longitudinal strain and strain rate using STE from the right parasternal four-chamber (RP4Ch) view, rather than the LAP4Ch view in dogs [18]. This approach provides a high quality four chamber image for STE analysis, avoiding the limits of LAP4Ch view (i.e., tracking errors due to breathing or side lobe artefacts, especially for LV apex and free wall) [18]. However, the authors of that study reported that a suboptimal visualization of the apical segments during the cardiac cycle from the RP4Ch view can induce tracking errors and data obtained from this view and LAP4Ch view were not interchangeable [18]. As the authors stated [18], RP4Ch is often more easily obtained than the LAP4Ch view in dogs; this is true also for feline patients.

To the best of the authors’ knowledge, no studies exist comparing longitudinal strain and strain rate values using STE from different echocardiographic views in cats. Therefore, we examined the feasibility to obtain longitudinal strain and strain rate from the RP4Ch view in cats; moreover, we evaluated the agreement between RP4Ch and LAP4Ch views for assessment of LV longitudinal strain and strain rate and intra-observer variability.

## 2. Materials and Methods

We prospectively included in the study all cats presenting for echocardiographic examination at the Cardiology Service of the Veterinary Teaching Hospital of Perugia University between November 2019 and February 2020. Healthy cats were recruited from hospital staff, veterinary students and clients. Cats were considered healthy based on an unremarkable history, complete physical examination and conventional transthoracic echocardiography. Cats with persistent non-sinus arrhythmias were excluded. Diseased cats receiving cardiac medications were not excluded. The present study protocol was presented to the local Ethical Committee and was carried out in accordance with the Declaration of Helsinki. After presentation of the study to the Ethical Committee of the Perugia University, no ethical concern was retained and advised that Ethics Approval was not required.

### 2.1. Standard Echocardiography

Conventional echocardiography was performed by the same echocardiographer (D.C.) using an ultrasound unit equipped with multifrequency 4–11 MHz phased-array transducer (MyLab Class C, Esaote, Genova, Italy) and acquiring echocardiographic images from the right and left parasternal imaging planes. All cats underwent an echocardiographic examination without sedation and were positioned in right and then left lateral recumbency with continuous electrocardiographic recording. Two-dimensional and M-mode echocardiography was performed using recommended right parasternal long- and short-axis views to measure the chamber size and wall thickness [19,20]. Doppler echocardiography imaging was used for assessing valvular insufficiencies/stenosis, intracardiac shunts and left ventricular diastolic function [21,22].

### 2.2. Speckle Tracking Echocardiography

**Figure 1:**
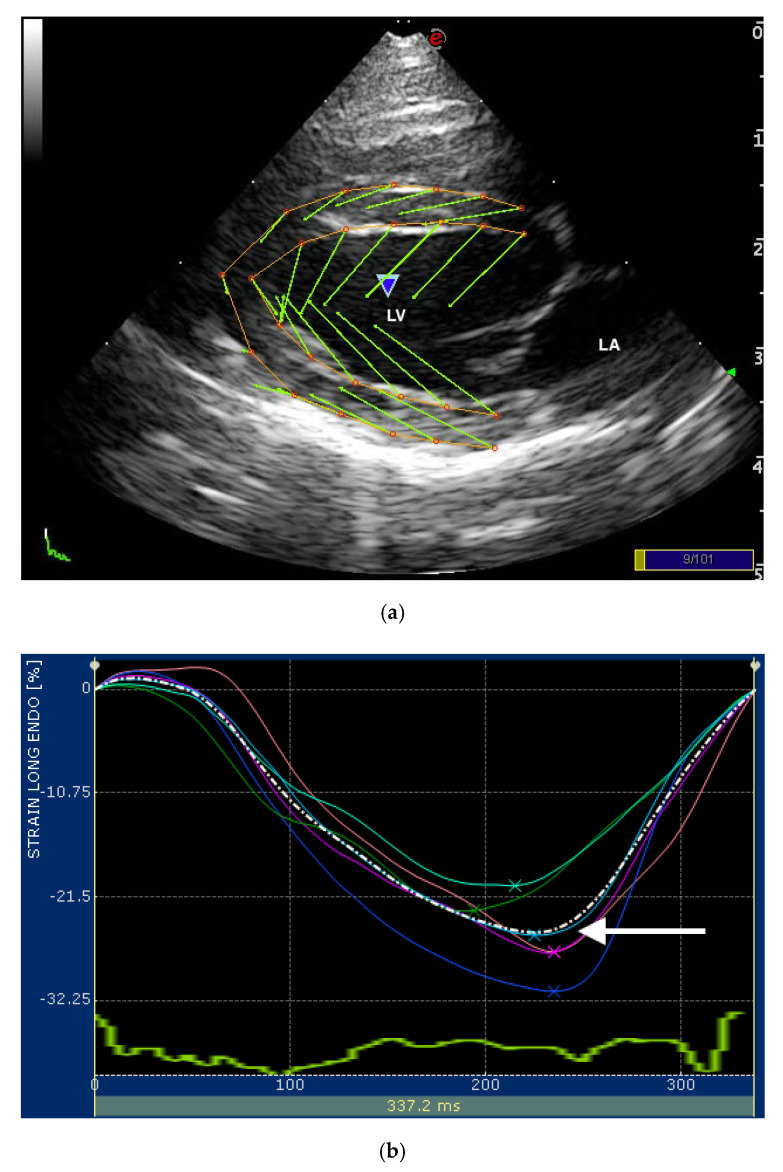

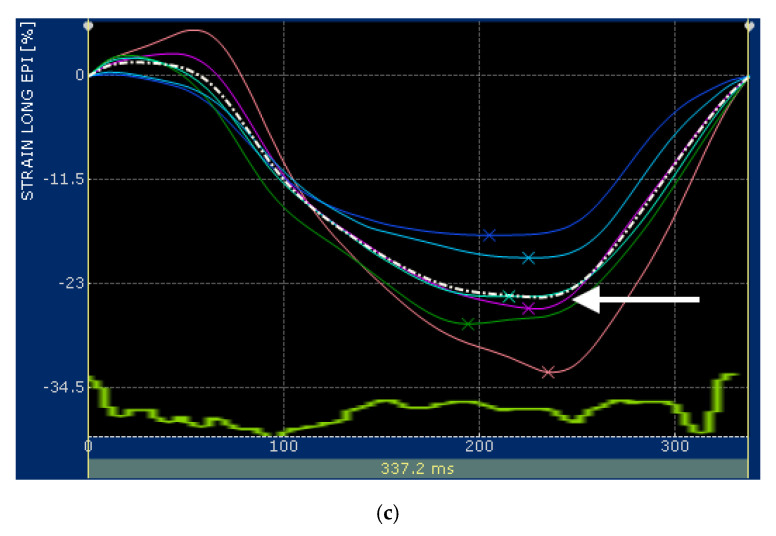
(**a**) Snapshot of 2-dimensional speckle tracking analysis from the right parasternal four-chamber view optimized for visualization of the left ventricle (LV) in a healthy cat. Thirteen points delimit and follow the endocardial and epicardial border of the LV, frame-by-frame during entire cardiac cycle. Green arrows represent the vectors of each point and their direction of displacement; (**b**,**c**) snapshots of (**b**) endocardial and (**c**) epicardial longitudinal strain curves of the LV. The software divided the LV into 6 segments (colored line) and generates an average value (dotted line). Only the systolic peak (white arrows) of the longitudinal endocardial and epicardial strain of the LV were used. LV—left ventricle; LA—left atrium.

Images of the LV for STE were obtained from the RP4Ch and LAP4Ch views and three cineloops (at least three beats for each) from both echocardiographic views were acquired for off-line analysis. Both echocardiographic views were required for cats to be included in the analyses. The visualization of the LV was optimized obtaining the image with the longest long-axis dimension possible in both views. If necessary, we increased the sector width during the acquisition of the RP4Ch view. In this way, the foreshortening of the LV apex was avoided. All recorded cineloops were analyzed by one experienced examiner (D.C.) using the Xstrain^TM^ software (Esaote, Genova, Italy). A frame with an optimally visualized endocardial and epicardial borders were selected for both views. The software automatically traced 10 equidistant lines among 3 starting points fixed by the operator (2 at the mitral valve annulus and 1 at the LV apex) for delimiting both endocardial and epicardial borders. These 13 points followed the endocardial and epicardial border during entire cardiac cycle and the operator could check if tracking were optimal or the points needed to be manually adjusted (Figure 1). The software divided the LV into 6 segments and generated an average value of the longitudinal strain and strain rate. Only average value of the maximal systolic peak of the LV longitudinal strain and strain rate of the endocardial and epicardial border for both views were analyzed (Figure 1). The mean value of three consecutive cardiac cycles in sinus rhythm for each variable was obtained and used for statistical analysis.

To assess the intra-observer measurement variability, ten echocardiograms of ten different cats were randomly selected from stored images for repeated measurements. Intra-observer measurement variability was obtained from each view analyzing the same cardiac cycles a second time at least 2 weeks after the initial examination.

### 2.3. Statistical Analysis

We compared heart rates during image acquisition in each echocardiographic view by a Wilcoxon Signed Ranks test.

We examined if the differences between the two echocardiographic views were normally distributed using a Shapiro–Wilk test. Because these were normally distributed, we compared the systolic peak of the LV longitudinal strain and strain rate values of endocardial and epicardial border between the two echocardiographic views using Limits of Agreement analysis for all cats [23].

We determined the intra-observer measurement variability for each view by calculating the percent difference between measurements by the same observer, as previously reported [8,9,10,24].

All analyses were performed using commercial statistical software (MedCalc Statistical Software version 19.1.5 (MedCalc Software bv, Ostend, Belgium; https://www.medcalc.org; 2020).

## 3. Results

We included 50 client-owned cats in the study: 31 clinically healthy cats and 19 with different disease states. The breeds of the cats included in the study consisted of domestic shorthair (47), Siamese (1), Persian (1) and Maine coon (1). Of the 50 cats, 20 were females and 30 males, with a median age of 60 months (range: 6 months–18 years) and weighing a median of 4 kg (range: 2–9 kg). The median heart rate recorded during the RP4Ch STE acquisition (191 beats/minute; range: 130–243 beats/minute) did not differ from that recorded during the LAP4Ch STE acquisition (187 beats/minute; range: 119–244 beats/minute; *p* = 0.66). Of the 19 diseased cats, 15 had cardiomyopathy and 4 had extracardiac disease. Of the cats with cardiomyopathy, 11 cats had hypertrophic cardiomyopathy, 2 cats had restrictive cardiomyopathy and 2 cats had unclassified cardiomyopathies. Extracardiac diseases included lungworm infestation (2 cats), chronic kidney disease (1 cat) and diabetes mellitus (1 cat).

We obtained the systolic peak of the LV longitudinal strain and strain rate values of endocardial and epicardial border from the RP4Ch and Lap4Ch views in all cats (Table 1).

**Figure 2:**
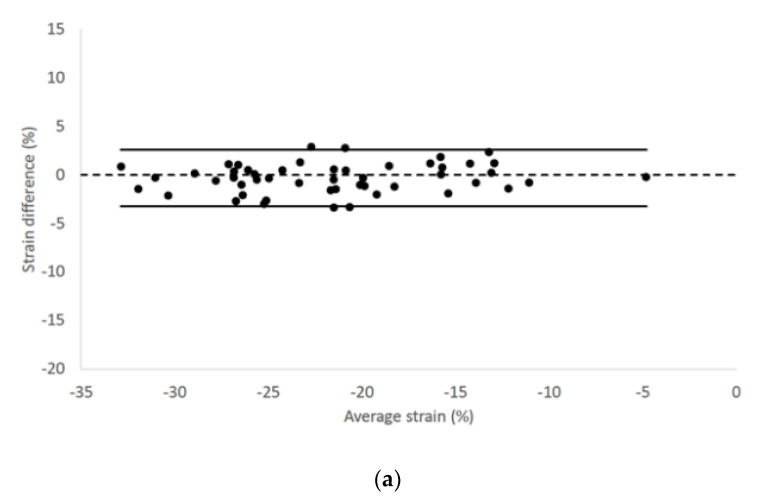

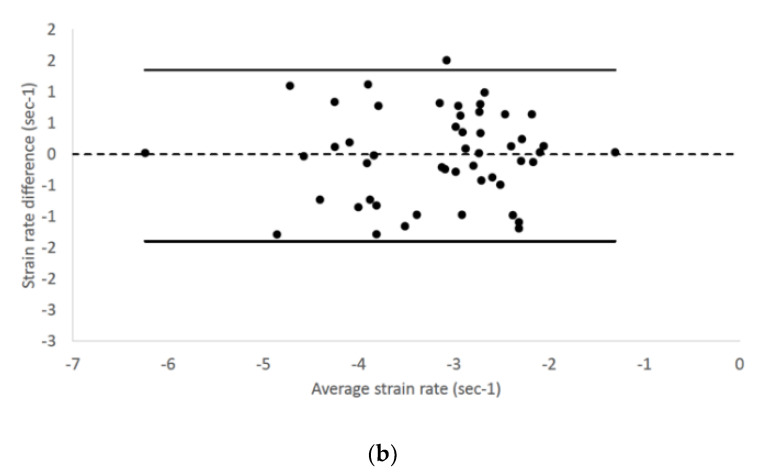
Bland–Altman plots for (**a**) endocardial longitudinal strain and (**b**) strain rate of all cats included in the study. The difference of the (**a**) endocardial longitudinal strain and (**b**) strain rate measurements obtained from right parasternal four-chamber and left apical four-chamber views are plotted against average measurements from both views. The dotted line represents zero difference between the two measurements; the solid lines represent the 95% limits of agreement between the two measurements.

We found no fixed or proportional bias when comparing LV longitudinal strain and strain rate values of endocardial and epicardial border obtained from the two different echocardiographic views. Endocardial longitudinal strain and strain rate had 95% limits of agreement from −3.28 to 2.58 and −1.41 to 1.36, respectively (Figure 2).

**Figure 3:**
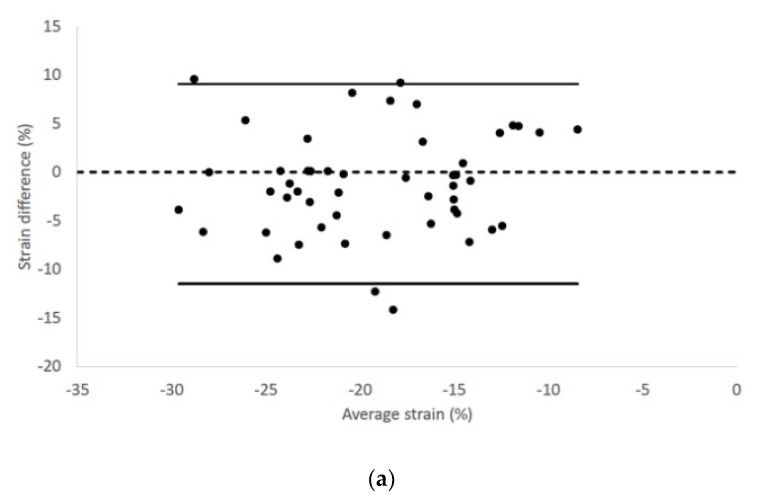

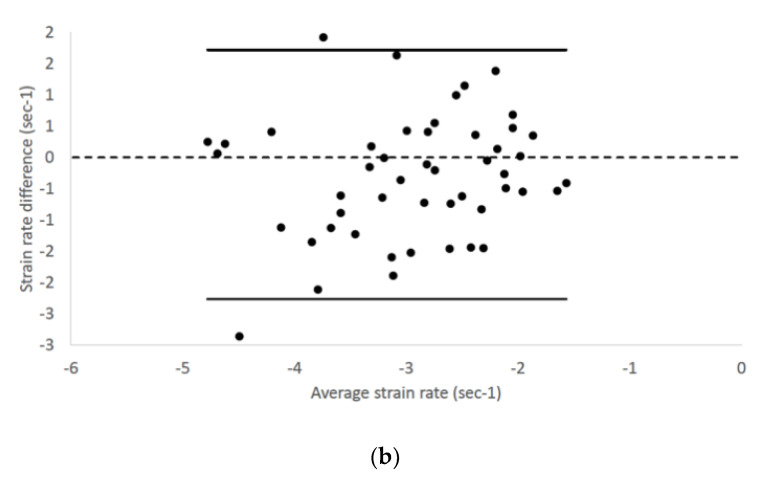
Bland–Altman plots for (**a**) epicardial longitudinal strain and (**b**) strain rate of all cats included in the study. The difference of the (**a**) epicardial longitudinal strain and (**a**) strain rate measurements obtained from right parasternal four-chamber and left apical four-chamber views are plotted against average measurements from both views. Dotted line represents zero difference between the two measurements; the solid lines represent the 95% limits of agreement between the two measurements.

Epicardial longitudinal strain and strain rate had 95% limits of agreement from −11.58 to 9.19 and −2.28 to 1.74, respectively (Figure 3).

Intra-observer measurement variability from RP4Ch view was <10% for LV longitudinal strain values and <20% for LV longitudinal strain rate values. Intra-observer measurement variability of all LV STE variables for both echocardiographic views is listed in Table 2.

## 4. Discussion

Our study demonstrates that longitudinal LV strain and strain rate values obtained from the RP4Ch or LAP4Ch view are similar in healthy and diseased cats. Indeed, the methods show no fixed or proportional bias, suggesting that they provide similar estimates of longitudinal LV deformation. However, only endocardial longitudinal strain values obtained with the two views agreed sufficiently to be used interchangeably. Conversely, the agreement between the two echocardiographic views for epicardial longitudinal strain/strain rate and endocardial longitudinal strain rate values was insufficient to recommend interchangeability. Intra-observer measurement variability from RP4Ch view was acceptable for all STE variables.

STE is an advanced imaging technique useful to assess myocardial function by strain and strain rate analysis. This novel echocardiographic modality is angle-independent, therefore beam angles do not influence the deformation analysis. Recently, the feasibility for assessment of the longitudinal strain and strain rate using STE from the RP4Ch view has been described in dogs [18]. This approach allowed investigators to obtain a good quality four chamber image for STE analysis, but, as the authors reported, a suboptimal tracking of the apical segments, especially during the ventricular diastole, could cause an inadequate analysis [18]. These authors concluded that data obtained from RP4Ch and LAP4Ch views were not considered interchangeable [18]. Our findings differed from those of the previous investigators; indeed, apical segments of the LV were easily visualized, and we could track the points used to delineate the endocardial and epicardial borders. Moreover, the software followed the myocardium during the entire cardiac cycle and the LV apex did not go out of the sector scan. We speculate that this different with previous study in dogs may be explained by the cardiac dimensions and orientation of the heart within the chest in cats compared to the dogs. Commonly, the LV was entirely visualized in the sector scan by RP4Ch view during the entire cardiac cycle in cats. On the other hand, in dogs, especially large breeds, the LV apex can move out of the scan sector in RP4Ch view.

A complete echocardiographic examination includes the visualization of both RP4Ch and LAP4Ch views in cats [18]. The LAP4Ch view is commonly used for LV Doppler and tissue Doppler imaging evaluations because an optimal alignment with blood flow or mitral annulus can be obtained. Other investigators have examined STE analysis of the LV from LAP4Ch view in cats [13,14,15,16]. However, RP4Ch view is often easier to obtain than the LAP4Ch view and should be considered when LAP4Ch view is suboptimal. Our results showed that strain and strain rate values were similar between the two echocardiographic views, but only endocardial longitudinal strain values were interchangeable. Contrary to the previous study in dogs [18], we could observe a difference between endocardial and epicardial deformation analysis because our software permitted separate evaluation of these components of the LV strain and strain rate.

Some authors have reported that RP4Ch view cannot include the true apex of the LV in dogs [25]; however, similar problems occur in humans from the LAP4Ch view [26]. Recently, several studies have reported that foreshortening of the LV in RP4Ch view was not evident if the view was imaged correctly in dogs [27,28,29,30]. In our study, to avoid a false visualization of the LV apex and a shortened LV long axis, we took care to obtain a LV image in both views with the longest possible long-axis dimension.

STE analysis requires high echocardiographic image quality to follow the speckles frame-by-frame during the cardiac cycle. Our software easily and consistently followed the epicardial and endocardial borders during the cardiac cycle in both echocardiographic views. However, although epicardial deformation variables and endocardial strain rate showed similar values using different echocardiographic views, we did not consider them interchangeable, because we deemed the absolute differences or the percentage differences too large. This may suggest that tracking of the epicardial border is suboptimal compared to endocardial border, possibly because of interference from pericardium or lungs. Moreover, whether or not STE has a certain intrinsic angle-dependence which can influence strain and strain rate values in different views, remains undetermined.

Intra-observer measurement variability showed low values for strain analysis in both echocardiographic views, while higher intra-observer measurement variability was observed for strain rate analysis. These findings agree with our previous STE studies of left atrium and right ventricle [8,9,10]; therefore, we cannot exclude that this high variability for some variables may be software dependent.

Our study has some limitations. We have evaluated only the global performance of the LV (average strain and strain rate of all segments, while in the previous study in dogs [18], the investigators analyzed and compared individual myocardial segments. However, veterinary clinicians are mostly interested in assessing global strain and strain rate, rather than regional dyskinesis, because myocardial infarction occurs infrequently in dogs and cats. Furthermore, we performed the strain and strain rate analysis using Xstrain^TM^ software, so our findings cannot be generalized to other STE software. Indeed, data obtained from software of different vendors are not interchangeable [31]. Finally, in our study population no cats with dilated cardiomyopathy or end-stage hypertrophic cardiomyopathy were present. The dilated LV, typically observed in these cardiomyopathies’ forms, could not permit to visualize the LV apex within the sector scan from RP4Ch view.

## 5. Conclusions

Our study demonstrates for the first time that longitudinal LV strain and strain rate can be obtained from the RP4Ch view in cats using the Xstrain^TM^ software. Clinicians can use the RP4Ch view to assess longitudinal LV deformation, but only endocardial longitudinal strain values agree sufficiently with those derived from LAP4Ch view to be used interchangeably.

## Figures and Tables

**Table 1:** Left ventricular longitudinal strain and strain rate values for endocardial and epicardial border obtained from right parasternal four-chamber (RP4Ch) and left apical four-chamber view (LAP4Ch) views in 50 cats included in the study.

STE Variables	RP4Ch View	LAP4Ch View
Endocardial LV longitudinal strain (%)	−21.71 ± 6.22 −22.23 (−32.68–−4.93)	−21.36 ± 5.98 −21.53 (−33.30–−4.70)
Epicardial LV longitudinal strain (%)	−19.83 ± 6.11 −21.02 (−31.56–−6.24)	−18.63 ± 5.55 −19.13 (−33.62–−9.68)
Endocardial LV longitudinal strain rate (Sec^−1^)	−3.19 ± 0.99 −2.88 (−6.23–−1.29)	−3.16 ± 0.96 −3.05 (−6.25–−1.32)
Epicardial LV longitudinal strain rate (Sec^−1^)	−3.08 ± 1.02 −2.92 (−5.92–−1.51)	−2.81 ± 0.90 −2.80 (−4.90–−1.36)

Data are presented as average ± standard deviation and median (max and min values). STE, speckle tracking echocardiography; RP4Ch, right parasternal four-chamber view; LAP4Ch, left apical four-chamber view; LV, left ventricular.

**Table 2:** Intra-observer measurement variability of the LV STE variables obtained from the RP4Ch and LAP4Ch views in 10 cats.

STE Variables	RP4Ch View	LAP4Ch View
Endocardial LV longitudinal strain	5.4	7.3
Epicardial LV longitudinal strain	8.2	13
Endocardial LV longitudinal strain rate	18.7	15.3
Epicardial LV longitudinal strain rate	17.5	19.2

Data presented as the average percentage difference (% difference = difference/mean × 100) for each STE variable. STE, speckle tracking echocardiography; RP4Ch, right parasternal four-chamber view; LAP4Ch, left apical four-chamber view; LV, left ventricular.
